# A Few Other Charities

**Published:** 1892-12-24

**Authors:** 


### A FEW OTHER CHARITIES.

**British Home for Incurables**, Clapham. (Office, 73, Cheapside, E.C.)—Helpless! Hopeless!! Homeless!!! These pathetic words form the motto of the British Home for Incurables, at Clapham, which national charity provides for the nursing and comfort, but *not expensive luxury*, of the suffering inmates of the Home. The board of management earnestly appeal for 500 new annual subscribers of one guinea each to counterbalance the loss of income sustained by selling out capital to build the new home now in course of erection at Streatham. The charity has nearly 300 pensioners, each receiving £20 a-year. Applications are constantly being received from all parts of the United Kingdom. Cheques and Postal Orders to be crossed "Barclay and Co.," and sent to the Secretary, Mr. Robert G. Salmond.

**City of London Truss Society**, 35, Finsbury Square.—For the relief of the ruptured poor throughout the kingdom. At the present time about 10,000 of both sexes and all ages are treated annually, while over 500,000 patients have been relieved since its formation eighty-five years ago. Secretary, Mr. John Whittington.

**The Hampstead Home Hospital and Nursing Institute** has made steady progress during the eleven years of its existence. Commencing with one house, it now comprises a block of four houses, with a separate house, connected by a covered way, for the private nursing staff. During the past year nearly 200 patients have been treated, in addition to a large number of emergency cases. This Institution deserves more local support, as the majority of the patients only pay a nominal fee, all accidents being admitted free.

**Home for Little Boys**, Farningham, Swanley, Kent. —An institution whose object is to receive all little lads, whether orphan or not, who, being homeless and destitute, are likely to fall into crime from lack of a friendly hand to save them. About 500 boys are provided for by this Institution in their homes at Farningham and Swanley, where they are trained from an early age till they become sufficiently expert at one of the many trades taught here to enter on life's duties fully equipped for their part in it. We commend this institution to the notice of those who have little lads of their own. Secretary, Mr. B. Clarke, Bank Buildings, Ludgate Circus, E.C.

**Invalid Children's Aid Association**, 18, Buckingham Street, Strand, W.C.—To aid in every way that it is possible the invalid and crippled children of the poor in London is the aim of this Association, and up to the present, so far as its resources have permitted, it has endeavoured to carry out this worthy aim. Its *modus operandi* is, first, to find each child a friend, who shall give it, so far as circumstances permit, unstinted personal service. This being done, skilled nursing, medical advice, surgical appliances, admission into hospitals and homes, are often required, and here again the Society steps in, and as far as possible makes the necessary provision. With such worthy aims, we think liberal assistance should be forthcoming this Christmas. Subscriptions should be sent to the Hon. Secretaries.

**Irish Distressed Ladies' Fund**, 17, North Audley Street, W.—The Committee appeal for funds to enable them to help those ladies of Ireland who depend for their support on the proceeds of Irish property, but who, owing to the rent difficulty and causes beyond their own control, have been reduced to absolute poverty. Relief is given independently of any question of politics or religion; employment is found, when possible, for those able to work; pecuniary help is given to the aged and infirm; and the education of children is in cases paid for. Secretary, Lieut.-General W. M. Lees.

**London Orphan Asylum**, Watford.—This institution for the maintenance and education of fatherless children of both sexes, is dependent upon nine-tenths of its income upon voluntary subscriptions, and we trust that it may meet with the cordial recognition it deserves. Since the charity was founded in the year 1813, five thousand five hundred and thirty-three fatherless children from all ranks in life, and from all parts of the kingdom have received the benefits of the institution. These admissions show an average of seventy additional children each year in the past seventy-nine years. Eighty-eight orphans have been received this year. Forty more are to be admitted in January. Five hundred and seven children are now in the asylum. The managers invite the sympathy and liberal support of the public, so that the work of the institution may continue with unabated vigour. £2,000 will be required to close the year free from debt. Contributions will be gratefully received. Secretary, Mr. James Rogers, 21, Great St. Helen's, E.C.

**Metropolitan Drinking Fountain and Cattle Trough Association.**—The relief afforded to both human beings and dumb animals by this Society is incalculable, as it is the only one which provides free supplies of water for man and beasts in the streets of London. It is entirely dependent on voluntary contributions. Secretary, Mr. M. W. Milton, 70, Victoria Street, Westminster, S.W.

**Mrs. Hilton's Crèche**, 12, 14, and 16, Stepney Causeway, E.—For nearly twenty years this most useful institution has been steadily working its way amongst the poor classes of the East-end, and supplying a long-felt need. Receiving and tending some eighty to a hundred children of all ages daily, it at once benefits both the child and the hard-working and poverty-stricken mother, who (unable, through poverty, illness, or the necessities of her daily toil, to tend her child during the day, can leave it in safety in this friendly shelter. The children, whose ages vary from three weeks to five years, are washed, clothed, fed, and tended for twelve hours each day for the nominal charge of twopence a-day. Mrs. Hilton's Crèche, whilst it relieves the parent of the burden of its care, lays the foundation of better health in the child by providing good food and careful nursing. Contributions should be sent to Mrs. Hilton, 12, Stepney Causeway.

**National Health Society.**—The basis of the whole system of instruction which this most useful Society comprises, may be said to lie in the proverb that "Prevention is better than Cure." It goes straight to the root of the matter, as its aim is to diffuse by all possible means sanitary knowledge of every kind, comprising home nursing, rearing of infants, prevention of the spread of infectious diseases, and by no means least, important lectures on sanitary reform in food and cookery. The Society gives courses of lectures on the various subjects which affect health, not only in London and its vicinity, but even so far afield as Glasgow and Aberdeen. We wish this useful Society all success in its work. Secretary, Miss Lankester, 53, Berners Street, W.C.

**National Society for the Prevention of Cruelty to Children**, central office, 7, Harpur Street, Bloomsbury, London, W.C.—The sole object of the society is to secure to every little English child that its life shall be at least endurable. Its motive is compassion for the unjust and wholly needlessly inflicted weaknesses and sorrows of the helpless young. By what it has done it has laid under obligation the gratitude of all truly manly and womanly hearts. It has stopped or ameliorated the needless pains and tears of 56,615 children. Parts of England, Ireland, and Wales are under the care of the society, and it seeks funds to enable it to include the whole. It works by the law which it passed; but its first consideration is not the law, but the child. Its object is not to provide homes for destitute children, but prisons for those who make them destitute. Where fear of courts suffices to stop a child's sufferings, it does not prosecute. But they must be stopped. It is awaking long-neglected natural instincts in careless and savage parents, and is teaching them to treat children properly. There is no work more hopeful for children, and none more likely to prove a blessing to the land. Unhappily, however, it has no less a debt than £4,500. Its expenditure is at the rate of £40,000 a year. Benjamin Waugh, hon. director.

**Orphan Working School,** 73, Cheapside, E.C. Founded 1758.—This is a national and unsectarian institution which maintains 600 children, varying in age from infancy to fourteen years. It is greatly in want of funds at the present time, as £5,000 is needed to pay off the debt to the bankers. This orphanage is the oldest one of the kind in the metropolis, and over 90 per cent. of the old scholars turn out satisfactorily. Secretary, Algernon P. Coote.

**Zenana, Bible, and Medical Mission,** 2, Adelphi Terrace, W.C.—This society has a threefold object in its work, addressing itself to the spiritual, mental, and bodily evils under which the unhappy women of India have groaned or centuries. It is worthy of help from all. Especially should it appeal to women, for their sisters in India can only be reached by women; their sufferings only alleviated by women; and only women can enter their homes and personally influence them in mind and body. The great need of the mission at the present time is for friends of the work to subscribe £25,000 to enable the Council to consolidate the Zenana school and medical work of this useful society. So energetic is the society that we hope the generosity of English men and women will enable them to do much more than it is possible for it to attempt with the present inadequate income. Hon. Finance Secretary, Mr. W. T. Paton.

The following are deserving institutions to the work of which we would also call the attention of the charitable :—

### General Hospitals.

French Hospital, 172, Shaftesbury Avenue, W.C.—Secretary, M. F. Sorel.

London Temperance Hospital, Hampstead Road, N.W.—Secretary, Mr. E. W. Taylor; Matron, Miss S. E. Orme.

### Consumption.

Royal National for Consumption, Ventnor.—Secretary, Mr. E. Morgan, 34, Craven Street, W.C.; Lady Secretary, Miss Moore.

### Children.

Alexandra Hospital for Children, 18, Queen Square, Bloomsbury, W.C.—Matron, Miss L. E. Moore.

Belgrave Hospital for Children, 79, Gloucester Street, Pimlico.—Hon. Secretaries, the Rev. J. Storrs and Mr. Percy Gates; Lady Superintendent, Miss Munro.

Cheyne Hospital, 46, Cheyne Walk, S.W.—Honorary Secretary, Mr. Reginald Blunt; Matron, Miss Elam.

Home for Incurable Children, 2, Maida Vale, W.—Matron, Miss Coleman.

### Fever.

London Fever Hospital, Liverpool Road, N.—Secretary, Major W. Christie; Matron, Miss H. F. Ingall.

### Skin.

London Skin Hospital, 47, Cranbourne Street, Leicester Square, W.C.—Secretary, Mr. H. A. Gifford.

### Dental.

Dental, 40, Leicester Square, W.C.—Secretary, Mr. F. J. Pink.

National Dental, 149, Great Portland Street.—Secretary, Mr. Arthur G. Klugh.

### Ophthalmic.

Central London, 238, Gray's Inn Road.—Secretary, Mr. William Abrams; Matron, Mrs. Hugall.

Royal Westminster, King William Street, W.C.—Secretary, Mr. T. Beattie-Campbell; Matron, Miss Hordern.

Western, 153, Marylebone Road.—Secretary, Mr. G. E. Manton.

### Lying-in.

British Lying-in, Endell Street, W.C. — Secretary, Mr. Fitzroy Gardner; Matron, Mrs. E. Freeman.

General Lying-in, York Road, Lambeth, S.E. — Secretary, Miss Annie Whyte; Matron, Miss Atkinson.

Queen Charlotte's Lying-in Hospital, Marylebone Road, N.W. —Secretary, Mr. G. Owen Ryan.

### A Few Other Charities.

Field Lane Refuges and Ragged Schools, Vine Street, Clerkenwell Road, E.C.—Secretary, Mr. Peregrine Platt.

Ragged School Union.—Secretary, Mr. John Kirk, Exeter Hall, W.C.

Religious Tract Society, 56, Paternoster Row, E.C.—Secretaries, Rev. L. B. White and Rev. S. Green.

Royal Albert Orphan Asylum, Bagshot, Surrey. — Secretary, 62, King William Street, E.C.

Royal Society for the Prevention of Cruelty to Animals, 105, Jermyn Street, S.W.—Secretary, Mr. John Colam.

Thames Church Mission, 31, New Bridge Street.—Secretary, Rev. H. Bloomer.

